# New variant and expression studies provide further insight into the genotype-phenotype correlation in *YAP1*-related developmental eye disorders

**DOI:** 10.1038/s41598-017-08397-w

**Published:** 2017-08-11

**Authors:** R. Holt, F. Ceroni, D. A. Bax, S. Broadgate, D. Gold Diaz, C. Santos, D. Gerrelli, N. K. Ragge

**Affiliations:** 1 Faculty of Health and Life Sciences, Oxford Brookes University, Oxford, UK; 2 Nuffield Department of Clinical Neurosciences, University of Oxford, Oxford, UK; 3 Institute of Child Health, University College London, London, UK; 4 Clinical Genetics Unit, West Midlands Regional Genetics Service, Birmingham Women’s and Children’s NHS Foundation Trust, Birmingham, UK

## Abstract

*YAP1*, which encodes the Yes-associated protein 1, is part of the Hippo pathway involved in development, growth, repair and homeostasis. Nonsense *YAP1* mutations have been shown to co-segregate with autosomal dominantly inherited coloboma. Therefore, we screened *YAP1* for variants in a cohort of 258 undiagnosed UK patients with developmental eye disorders, including anophthalmia, microphthalmia and coloboma. We identified a novel 1 bp deletion in *YAP1* in a boy with bilateral microphthalmia and bilateral chorioretinal coloboma. This variant is located in the coding region of all nine *YAP1* spliceforms, and results in a frameshift and subsequent premature termination codon in each. The variant is predicted to result in the loss of part of the transactivation domain of YAP1, and sequencing of cDNA from the patient shows it does not result in nonsense mediated decay. To investigate the role of *YAP1* in human eye development, we performed *in situ* hybridisation utilising human embryonic tissue, and observed expression in the developing eye, neural tube, brain and kidney. These findings help confirm the role of *YAP1* and the Hippo developmental pathway in human eye development and its associated anomalies and demonstrate its expression during development in affected organ systems.

## Introduction

Developmental eye anomalies, including anophthalmia (absent eye), microphthalmia (small eye) and coloboma (gap in the eye structure) (AMC) are a genetically heterogeneous group of disorders affecting 11.9 per 100,000 live births^1^. Increasing numbers of causative genes have been identified for these anomalies, including *SOX2* ^2^, *OTX2* ^3^, *BMP4* ^4^, *BMP7* ^5^ and *STRA6* ^6^. Recently, Williamson *et al*. reported two large pedigrees each containing multiple individuals with developmental eye anomalies which were inherited in an autosomal dominant fashion. In both families they identified co-segregating heterozygous nonsense mutations in *YAP1* ^7^. In addition to the eye anomalies, affected members of one family also exhibited variable extraocular features including hearing loss, intellectual disability, haematuria and orofacial clefting^7, 8^. Further screening of *YAP1* in a large cohort of individuals with eye anomalies identified an additional four variants classified as of unknown pathogenicity for various reasons: being inherited from an unaffected parent (1 case), parental DNA being unavailable (2 cases), or as the mutation was a copy number variant affecting multiple genes, including *YAP1* (1 case)^7^. Similarly, Oatts *et al*. identified a novel heterozygous mutation in a family with coloboma and microphthalmia with evidence of incomplete penetrance^9^. Finally, Fossdal *et al*. identified a mutation of TEAD1, a cofactor of YAP1, as a cause of helicoid peripapillary chorioretinal degeneration. The mutation occurs in a potential binding site for YAP1, leading them to postulate that the change alters the ability of YAP1 to bind TEAD1, affecting the expression of other genes^10^.

**Figure 1:**
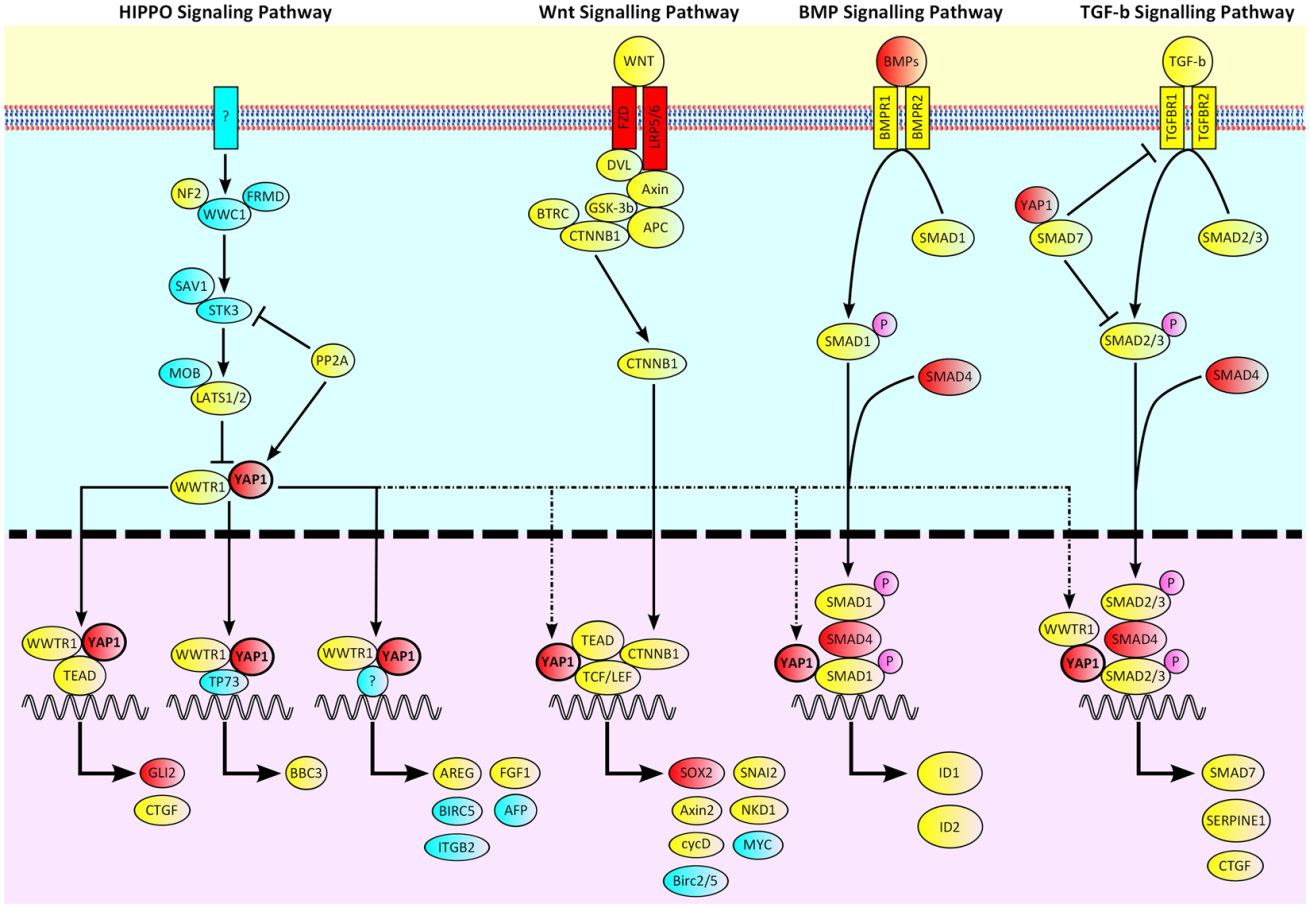
Cartoon illustrating the pathways with which YAP1 interacts. Known anophthalmia, microphthalmia and coloboma genes are coloured red. Genes implicated in eye function are coloured yellow. Genes not yet implicated in eye development or function are coloured blue.

YAP1 is a transcriptional co-activator, and in combination with TAZ (encoded by *WWTR1*), is a major effector of the Hippo pathway to regulate organ size, binding TEAD1-4 to promote transcription^11, 12^ (Fig. 1). Activation of the Hippo pathway results in phosphorylation of YAP1 and TAZ, causing their migration from the nucleus to the cytoplasm, thus inhibiting their effect on transcription^11^. *YAP1* expression is positively correlated with that of *SOX2* ^11, 13^. Furthermore, reduction in *SOX2* expression also correlates with decreased expression of multiple targets of YAP/TEAD^11^. *YAP1* is directly regulated by SOX2, which is able to bind a CpG island 5′ of exon 1, as well as to exon 2^11, 13^. YAP1 also binds β-catenin and therefore may affect β-catenin-dependent Wnt signalling^13^. Zebrafish *Y* *a* *p* *1* mutants (*y* *a* *p* ^*nl13*/*nl13*^ and c.158_161del mutant lines, respectively) exhibit colobomas and loss of retinal pigment epithelium (RPE) in a fully penetrant fashion, but with varying extent and localisation, including asymmetric eye involvement in a single fish^14^. Typically, deficiency in RPE mainly occurs in the posterior part of the eye, but also affects the lateral and ventral surfaces^14^. Interestingly, medaka (Japanese rice fish) *Yap hir* mutants have a flattened body shape and mislocalisation of the lens to outside of the eye, due to loss of the filopodia normally tethering them to the retina. Therefore, Yap is essential for tissue tension and body shape in medaka^15^. *YAP1* and *TAZ* have also been shown to be expressed in the human adult cornea^16^. Furthermore, a large number of genes and proteins in the pathways with which YAP1 interacts have been implicated in AMC or eye function (Fig. 1).

Therefore, we screened *YAP1* in our UK cohort of patients with developmental eye anomalies to determine whether any potentially pathogenic variants were present.

## Results

### Patient screening

**Figure 2:**
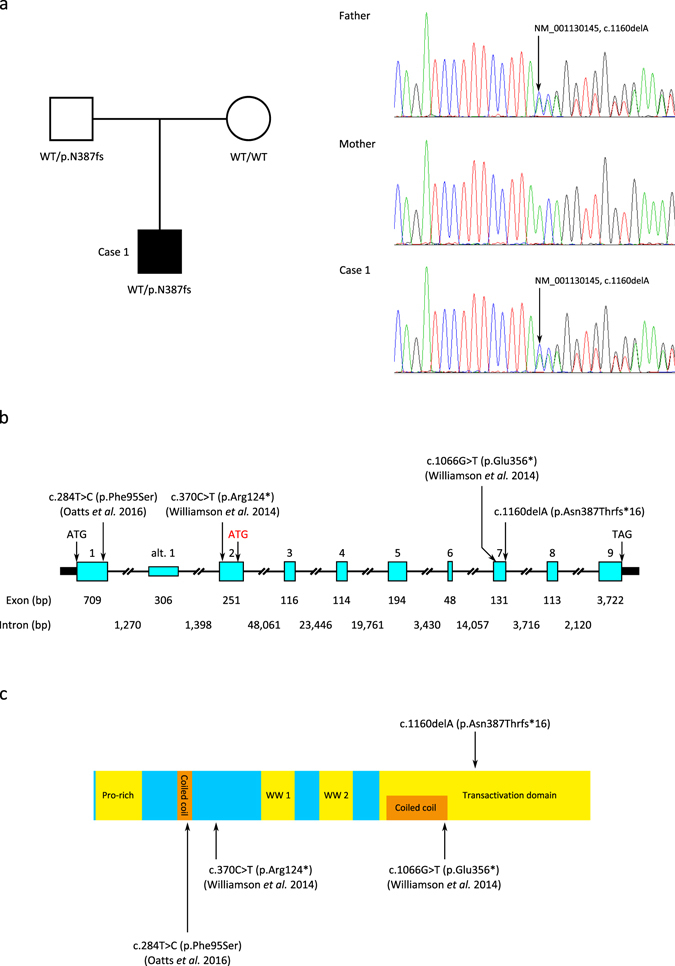
Identification of a novel *YAP1* frameshift mutation in a patient with developmental eye disorders. (**a**) The mutation is heterozygous in the patient and inherited from his asymptomatic father. (**b**) Schematic of the genomic structure of *YAP1*. Exons are indicated by boxes and are drawn to scale with the exception of the untranslated regions (shown as solid black boxes). Exon and intron sizes are shown beneath. Initiation and termination codons are indicated, with the alternate initiation codon of the NM_001195045.1 transcript indicated in red. The positions of the c.1160delA (p.Asn387Thrfs*16) reported here and the co-segregating nonsense mutations identified by Williamson *et al*.^7^ and the missense mutation identified by Oatts *et al*.^9^ are indicated. Alt. 1 = alternative exon 1 (NM_001195045.1 transcript). (**c**) The p.Asn387Thrfs*16 variant is located within the transactivation domain of YAP1 (NP_001123617). The locations of the co-segregating nonsense mutations identified by Williamson *et al*.^7^ and the missense mutation identified by Oatts *et al*.^9^ are also shown.

A UK cohort with ocular anomalies, principally anophthalmia, microphthalmia, and coloboma (AMC), was recruited as part of a national ‘Genetics of Eye and Brain anomalies’ study (REC study number 04/Q0104/129). In total, 258 UK patients were screened for variations in the coding region and flanking sequences of *YAP1* using LightScanner high resolution analysis^17^ with subsequent validation by Sanger sequencing. A novel heterozygous single base pair deletion variant was identified in exon 7 of case 1 (NM_001130145; exon7; c.1160delA; NP_001123617; p.Asn387Thrfs*16), observed by sequencing in both orientations. This variant was not present in the databases dbSNP147, ExAC^18^ or gnomAD^18^. Parental DNA samples were sequenced for both strands of exon 7, and the variant was identified in the heterozygous state in the asymptomatic father (Fig. 2). This variant occurs at chr11:102,223,749 (GRCh38/hg38) within the coding region of *YAP1*. There are nine known splice variants of *YAP1* encoding proteins of between 326 and 508 amino acids. All contain exon 7 and are predicted to have a frameshift in the presence of the variant, resulting in a premature termination codon, 16 codons after that in which the deletion occurs, leading to the loss of 118 amino acids that form part of the transactivation domain of the protein, the region required for the binding of other proteins. However, the premature termination codon is located 69 nucleotides upstream from the final exon-exon junction and therefore it could cause the nonsense mediated decay of all transcripts of *YAP1* containing it^19^. In addition, the c.1160delA occurs four nucleotides from the 3′ end of exon 7 and therefore could potentially affect splicing of the mRNA. Analysis of the mutation using the online tool Human Splicing Finder (http://www.umd.be/HSF3/index.html)^20^ indicated that the variant might alter *YAP1* splicing by alteration of an exonic splicing enhancer or suppressor site, or by activation of a cryptic donor site.

### *YAP1* expression in Case 1

To determine whether the c.1160delA variant does alter splicing or cause nonsense mediated decay, RNA was obtained from a Case 1 saliva sample using Oragene RNA kits and cDNA generated by reverse transcription. PCR of the cDNA for 45 cycles using forward and reverse primers in *YAP1* exons 7 and 9, respectively, resulted in a strong primary band of the expected size of 549 bp and a weak secondary band of approximately 350 bp. No bands were present in control PCRs using the products of reverse transcription reactions lacking reverse transcriptase, indicating the results were not due to genomic DNA contamination. Gel purification and sequencing of the stronger band revealed the presence of transcripts both with and without the c.1160delA variant, whereas sequencing for the secondary band failed. RNA was unavailable for either of Case 1’s parents. Thus, the mutation does not lead to either alternate splicing or nonsense mediated decay.

### Case description

The case presented is a 9 year old boy born via a normal delivery at 42 weeks’ gestation with a birth weight of 8 lb 6 oz. He has bilateral microphthalmia with a corneal diameter of 8mm on the right and 4mm on the left. This was associated with bilateral chorioretinal coloboma more marked on the left, bilateral posterior embryotoxon, nystagmus and a small left convergent squint. His developmental milestones were normal, but he was diagnosed with Asperger’s syndrome at age 5 years. His growth parameters are within normal range, although tracking along the lower centiles, with his height between the 2^nd^ and 9^th^ centile, his weight on the 25^th^ centile and his head circumference between the 2^nd^ and 9^th^ centile at 9 years of age. Urine analysis was normal. Karyotyping and array CGH results were normal. No other systemic abnormalities were observed. Both parents had a normal eye examination.

### *YAP1* expression during human development

**Figure 3:**
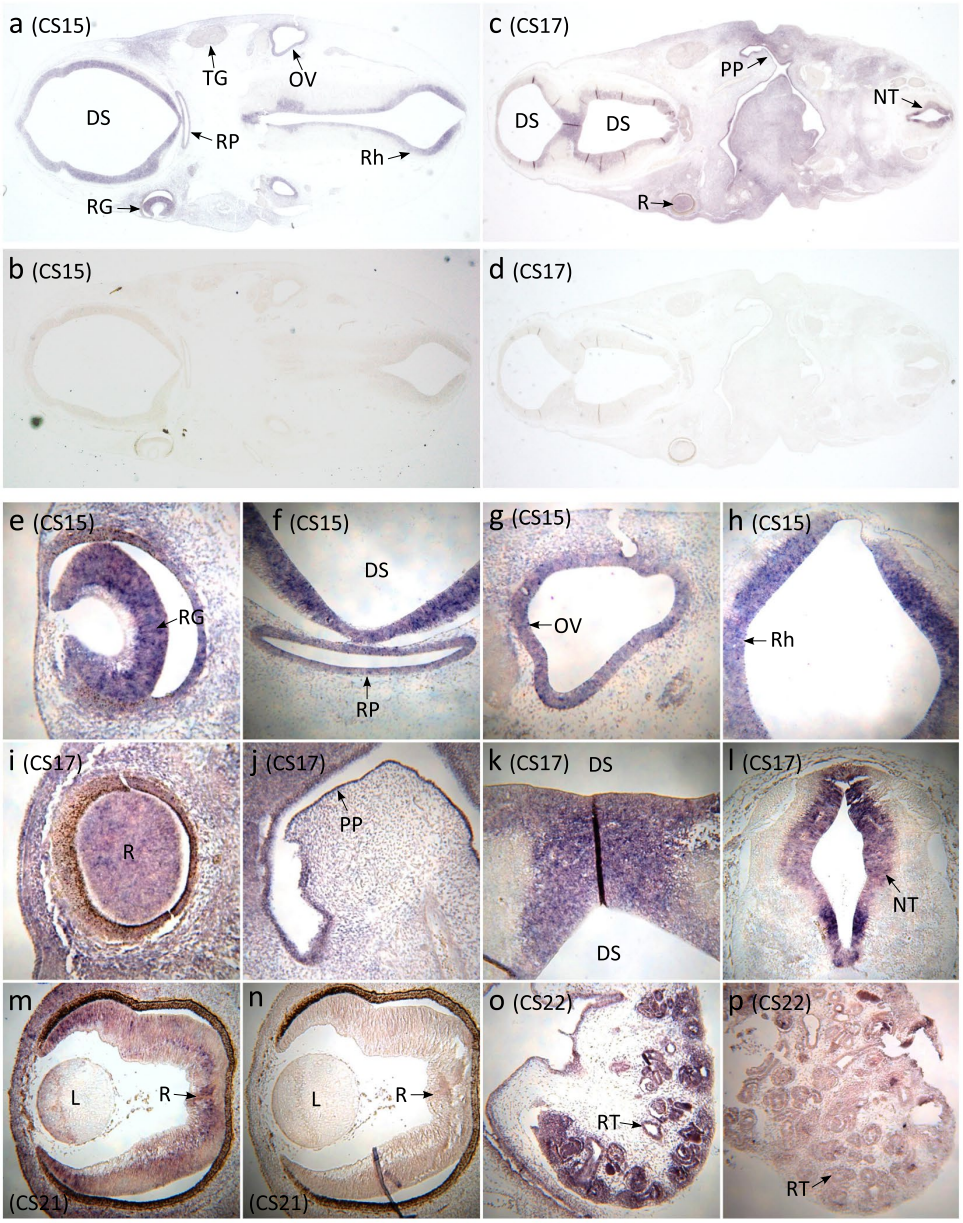
*YAP1 in situ* hybridisation studies in the developing human. (**a**,**b**) *In situ* hybridisations of coronal sections of a CS15 human fetus using *YAP1* antisense (**a**) and sense (negative control) (**b**) probes reveal expression in structures including the diencephalic superventricle (future third ventricle), retinal ganglion cell layer, Rathke’s pouch (future pituitary), trigeminal ganglion, otic vesicle, and rhombencephalon (future fourth ventricle). (**c**,**d**) *In situ* hybridisations of coronal sagittal sections of a CS17 human fetus using *YAP1* antisense (**c**) and sense (negative control) (**d**) show expression of *YAP1* in diencephalic superventricle, retina, primordium of the lateral palatine process, and neural tube. (**e–h**) High magnification images of *YAP1* expression in the CS15 human fetus in multiple structures: (**e**) retinal ganglion cell layer, (**f**) diencephalic superventricle and Rathke’s pouch, (**g**) otic vesicle, and (**h**) rhombencephalon. (**i–l**) High magnification images of *YAP1* expression in the CS17 human fetus in multiple structures: (**i**) retina, (**j**) primordium of the lateral palatine process, (**k**) diencephalic superventricle, and (**l**) the neural tube. (**m**,**n**) High magnification images of *YAP1* expression in the retina of CS21 human fetus: (**m**) antisense, and (**n**) sense. (**o**,**p**) High magnification images of *YAP1* expression in the kidney of CS22 human fetus: (**o**) antisense, and (**p**) sense. Abbreviations: DS = diencephalic superventricle; L = lens; NT = neural tube; OV = otic vesicle; PP = primordium of the lateral palatine process; R = retina; RG = retinal ganglion cell layer; Rh = rhombencephalon; RP = Rathke’s pouch; RT = renal tubules; TG = trigeminal ganglion.

As microphthalmia and coloboma are due to aberrations during eye development, we examined *YAP1* expression in human embryonic brain and eye. Furthermore, as haematuria has been reported in some patients with *YAP1* mutations^7, 8, 21^, we also examined expression in the human embryonic kidney. Nonradioactive RNA *in situ* hybridisation was performed on human embryo sections at Carnegie Stages (CS) 15, 17, 21 and 22^4^. Human embryos were obtained from the MRC/Wellcome Trust Human Developmental Biology Resource, UCL, with full ethical approval. *YAP1* expression was observed in multiple structures at CS15 (Fig. 3a,b,e–h). Of immediate relevance to the phenotype of Case 1 was expression in the developing eye, including the retina. Further expression was found in the ectoderm forming Rathke’s pouch, which advances during development to form the adenohypophysis (anterior pituitary). *YAP1* was also expressed in the otic vesicle (primitive ear), the region surrounding the ventricle of the diencephalon, which forms part of the forebrain, the tissue of the rhombencephalon (hindbrain), as well as the trigeminal ganglion. In contrast, by CS17 the most prominent expression was observed in the retina, diencephalic superventricle, neural tube, and the primordium of the lateral palatine process (Fig. 3c,d,i–l). Expression in the retina continued to be observed at CS21 (Fig. 3m,n), and was also observed in the renal tubules of the developing kidney at CS22 (Fig. 3o,p).

## Discussion

In this study we identify a novel frameshift mutation in a boy with bilateral microphthalmia and bilateral chorioretinal coloboma, inherited from his unaffected father. We show that this mutation does not affect splicing and does not lead to nonsense mediated decay of the mRNA. We demonstrate that *YAP1* is expressed in multiple structures in the developing human embryo, including the retina, brain and kidney.

Mutations in *YAP1* have been associated with both syndromic and non-syndromic forms of developmental eye disorders^7^. It has been suggested that this is related to whether the variant affects a single or multiple transcripts of the gene, with the former being related to the nonsyndromic form, and the latter to a syndromic phenotype^7, 9^. However, the p.Asn387Thrfs*16 frameshift reported here is located within exon 7 of *YAP1* and is predicted to affect all known splice forms, yet with the exception of Asperger’s syndrome the patient has no extraocular phenotype. This novel frameshift mutation was inherited from an asymptomatic parent. However, variable expressivity in relation to *YAP1* mutations has been previously reported in humans^7^, as well as in zebrafish models^14^. In addition, it is possible that the case contains a modifying mutation within a second gene which would account for the difference in phenotypes between father and child. Alternatively, it is possible that the mutation arose *de novo* in the father as a mosaic alteration, accounting for his lack of an AMC phenotype. Unfortunately, DNA was unavailable from the paternal grandparents to test this hypothesis. Our *in situ* data identified sequential expression of *YAP1* in relevant structures of the developing human eye, to our knowledge the first time this has been shown. Expression within the developing retina is consistent with the chorioretinal colobomas reported in Case 1, as well as those in previously described families^7, 9^. This is further supported by data showing that optic vesicle progenitor cells in zebrafish *Yap1* mutants lose their ability to form RPE^14^. Interestingly, given the presence of haematuria and deafness in one previously reported family with a *YAP1* nonsense mutation^7, 8, 21^, we also identified expression within the developing kidney and otic vesicle, consistent with its relevance to these additional phenotypes. The presence of Asperger’s syndrome in Case 1 is intriguing, given reports of intellectual disability in other families with *YAP1* alterations^7^ and our demonstration of *YAP1* expression in multiple regions of the developing human brain. However, due to the genetic complexity of autism spectrum disorders^22^, such correlations must still be approached with caution. Finally, the *YAP1* phenotypes observed in Case 1 and those previously reported are less severe than for mutations in *SOX2* ^2, 3, 7, 9, 23^. SOX2 is able to directly regulate *YAP1* ^13^, indicating that as a downstream gene the range of its effects is less, consistent with more moderate phenotypes. Therefore, this study contributes to the evidence for the role of *YAP1* mutations as a rare cause of developmental eye disorders and demonstrates its expression in multiple tissues during human development, highlighting the importance of screening this gene in patients with other phenotypes, including deafness and haematuria.

## Methods

### Cohort Description

A UK cohort with ocular anomalies, principally AMC, was recruited as part of a national ‘Genetics of Eye and Brain anomalies’ study with ethical approval from the Regional Ethics Committee Cambridge East (REC study number 04/Q0104/129). Participant informed consent was obtained according to the tenets of the Declaration of Helsinki. Patients were screened by indication in known AMC genes. From this cohort 258 individuals without a genetic diagnosis were screened for variants in *YAP1*. Of these, 32 individuals had bilateral anophthalmia or unilateral anophthalmia with contralateral microphthalmia, 43 had bilateral microphthalmia (+/−coloboma), 146 had unilateral anophthalmia/microphthalmia (+/−contralateral defects), and 37 had other phenotypes, including anterior segment dysgenesis. All subsequent experiments utilising the patient samples were performed in accordance with the relevant guidelines and regulations.

### Mutation Analysis

All nine coding exons of *YAP1* (NM_001282101.1), the alternate second exon of NM_001195045.1, and flanking sequences were screened for mutations by high resolution melting curve analysis on a LightScanner® (Idaho Technology Inc). PCRs for LightScanner melt curve analysis consisted of 10ng of template DNA, and final concentrations of 0.25 µM of each primer, 1x HotShot Diamond Master Mix (ClentLife) and 1x LCGreen Plus Dye (BioFire Diagnostics Inc) in a total volume of 10 µl. PCRs were typically one cycle of 5 minutes 95 °C, 45 cycles of 95 °C 30 s, annealing temperature 30 s and 72 °C 45 s, followed by a final denaturation step of 95 °C for 30 s. PCRs were performed in Framestar® 96 (4titude) 96-well plates with a 20 µl mineral oil overlay. Melt curves were generated on a LightScanner® (Idaho Technology Inc) using autoexposure, a starting temperature of 75 °C and a stop temperature of 98 °C. Data was analysed using the LightScanner® Instrument & Analysis Software (Idaho Technology Inc). Melt curves were normalised prior and post the major melt transition and aberrant curves detected using the autogroup function and manual inspection. Samples with an initial fluorescence of less than 600 were excluded from analysis, while those with a starting fluorescence less than 800 were not included during normalisation. Samples with aberrant melt curves were analysed using Sanger sequencing (primers available upon request) and sequence data analysed using CodonCode Aligner (CodonCode Corporation, Dedham, MA) or Chromas (Technelysium).

### RNA extraction and reverse transcription

RNA was extracted from saliva samples using Oragene® RNA kits (DNA Genotek) following the manufacturer’s instructions. Reverse transcription was performed using a QuantiTect Reverse Transcription kit (Qiagen) following the manufacturer’s instructions. Resulting RNA concentrations were determined using a NanoDrop^TM^ One (Nanodrop).

### cDNA PCR

PCRs consisted of 5 µl of template cDNA, and final concentrations of 0.25 µM of each primer, 1x HotShot Diamond Master Mix (ClentLife) in a total volume of 10 µl. PCRs were one cycle of 5 minutes 95 °C, 45 cycles of 95 °C for 30 s, 60 °C for 30 s and 72 °C for 45 s. The forward (AGTTACCAACACTGGAGCAG) and reverse (AAACTGCAACTGGCTTATGGA) primers were designed to anneal to exon 7 and the 3′ UTR of *YAP1*, respectively.

### PCR gel purification

PCR products were purified from agarose gels by band excision using a QIAquick Gel Extraction kit (Qiagen), following the manufacturer’s protocol.

### *In Situ* Hybridisation

Nonradioactive RNA *in situ* hybridisation was performed on human embryo sections at Carnegie Stages (CS) 15, 17, 21 and 22 as described elsewhere^4^. Probes were designed within the 3′ UTR of *YAP1* to bind all known splice variants and generated using primers GCTCGGCGGCCGCACTTGCTCCTACTTCTATGCTGA and GCTCGGTCGACGGCACTCCTTCCAAGTAGCT. Human embryos were obtained from the MRC/Wellcome Trust Human Developmental Biology Resource, UCL, with full ethical approval.

### Data Availability

No datasets were generated or analysed during the current study.

## References

[1] Shah SP (2011). Anophthalmos, microphthalmos, and typical coloboma in the United Kingdom: a prospective study of incidence and risk. Invest Ophthalmol Vis Sci..

[2] Fantes J (2003). Mutations in SOX2 cause anophthalmia. Nat Genet..

[3] Ragge NK (2005). SOX2 anophthalmia syndrome. Am J Med Genet A..

[4] Bakrania P (2008). Mutations in BMP4 cause eye, brain, and digit developmental anomalies: overlap between the BMP4 and hedgehog signaling pathways. Am J Hum Genet..

[5] Wyatt AW, Osborne RJ, Stewart H, Ragge NK (2010). Bone morphogenetic protein 7 (BMP7) mutations are associated with variable ocular, brain, ear, palate, and skeletal anomalies. Hum Mutat..

[6] Chassaing N (2013). Mutation analysis of the *STRA6* gene in isolated and non-isolated anophthalmia/microphthalmia. Clin Genet..

[7] Williamson KA (2014). Heterozygous loss-of-function mutations in YAP1 cause both isolated and syndromic optic fissure closure defects. Am J Hum Genet..

[8] Ravine D, Ragge NK, Stephens D, Oldridge M, Wilkie AO (1997). Dominant coloboma-microphthalmos syndrome associated with sensorineural hearing loss, hematuria, and cleft lip/palate. Am J Med Genet..

[9] Oatts JT (2016). Novel heterozygous mutation in YAP1 in a family with isolated ocular colobomas. Ophthalmic Genet..

[10] Fossdal R (2004). A novel TEAD1 mutation is the causative allele in Sveinsson’s chorioretinal atrophy (helicoid peripapillary chorioretinal degeneration). Hum Mol Genet..

[11] Basu-Roy U (2015). Sox2 antagonizes the Hippo pathway to maintain stemness in cancer cells. Nat Commun..

[12] Hauri S (2013). Interaction proteome of human Hippo signaling: modular control of the co-activator YAP1. Mol Syst Biol..

[13] Seo E (2013). SOX2 regulates YAP1 to maintain stemness and determine cell fate in the osteo-adipo lineage. Cell Rep..

[14] Miesfeld JB (2015). Yap and Taz regulate retinal pigment epithelial cell fate. Development..

[15] Porazinski S (2015). YAP is essential for tissue tension to ensure vertebrate 3D body shape. Nature..

[16] Raghunathan VK (2014). Involvement of YAP, TAZ and HSP90 in contact guidance and intercellular junction formation in corneal epithelial cells. PLoS One..

[17] Kennerson ML (2007). Mutation scanning the GJB1 gene with high-resolution melting analysis: implications for mutation scanning of genes for Charcot-Marie-Tooth disease. Clin Chem..

[18] Lek M (2016). Analysis of protein-coding genetic variation in 60,706 humans. Nature.

[19] Popp MW, Maquat LE (2016). Leveraging rules of nonsense-mediated mRNA decay for genome engineering and personalized medicine. Cell..

[20] Desmet FO (2009). Human Splicing Finder: an online bioinformatics tool to predict splicing signals. Nucleic Acid Res..

[21] Stephens D, Ravine D, Ragge N, Wilkie AOM (1997). Audiometric findings in a family with ophthalmological and renal disorders, intellectual disabilities and cleft lip and palate. J Audiol Med..

[22] Holt R, Monaco AP (2011). Links between genetics and pathophysiology in the autism spectrum disorders. EMBO Mol Med..

[23] Bakrania P (2007). SOX2 anophthalmia syndrome: 12 new cases demonstrating broader phenotype and high frequency of large gene deletions. Br J Ophthalmol..

